# Double Negative (CD3^+^4^−^8^−^) TCRαβ Splenic Cells from Young NOD Mice Provide Long-Lasting Protection against Type 1 Diabetes

**DOI:** 10.1371/journal.pone.0011427

**Published:** 2010-07-02

**Authors:** Beverly Duncan, Cristina Nazarov–Stoica, Jacqueline Surls, Margaret Kehl, Constantin Bona, Sofia Casares, Teodor-D. Brumeanu

**Affiliations:** 1 National Cancer Institute, National Institutes of Health, Bethesda, Maryland, United States of America; 2 Department of Medicine, Uniformed Services University of the Health Sciences, Bethesda, Maryland, United States of America; 3 Department of Microbiology, Mount Sinai School of Medicine, New York, New York, United States of America; 4 Naval Medical Research Center, Silver Spring, Maryland, United States of America; New York University, United States of America

## Abstract

**Background:**

Double negative CD3^+^4^−^8^−^ TCRαβ splenic cells (DNCD3) can suppress the immune responses to allo and xenografts, infectious agents, tumors, and some autoimmune disorders. However, little is known about their role in autoimmune diabetes, a disease characterized by the reduction of insulin production subsequent to destruction of pancreatic β-cells by a polyclonal population of *self*-reactive T-cells. Herein, we analyzed the function and phenotype of DNCD3 splenic cells in young NOD mice predisposed to several autoimmune disorders among which, the human-like autoimmune diabetes.

**Methodology/Principal Findings:**

DNCD3 splenic cells from young NOD mice (1) provided long-lasting protection against diabetes transfer in NOD/Scid immunodeficient mice, (2) proliferated and differentiated in the spleen and pancreas of NOD/Scid mice and pre-diabetic NOD mice into IL-10-secreting T_R_-1 like cells in a Th2-like environment, and (3) their anti-diabetogenic phenotype is CD3^+^(CD4^−^CD8^−^)CD28^+^CD69^+^CD25^low^ Foxp3^−^ iCTLA-4^−^TCRαβ^+^ with a predominant Vβ13 gene usage.

**Conclusions/Significance:**

These findings delineate a new T regulatory component in autoimmune diabetes apart from that of NKT and CD4^+^CD25^high^ Foxp3^+^T-regulatory cells. DNCD3 splenic cells could be potentially manipulated towards the development of autologous cell therapies in autoimmune diabetes.

## Introduction

A unique population of T regulatory cells other than Foxp3^+^ CD4^+^ and IL-10-secreting T_R_-1 cells was recently described as “natural suppressors” CD3^+^4^−^8^−^/TCRαβ double negative T-cells (DNCD3 cells). These cells were first described in the spleen of adult mice [1], and human peripheral blood [2]. DNCD3 cells represent about 1–3% of the peripheral lymphocytes in mice and humans. Phenotypic analysis showed that an important fraction of DNCD3 cells lack expression of NK1.1 and B220 antigens, and express a CD44^hi^ memory-like phenotype [3], [4]. Analysis of TCR α and β families of DNCD3 splenocytes in healthy mice and human subjects revealed a broad TCR Vβ repertoire [2], [5]. Yet, the TCRαβ repertoire of DNCD3 splenic cells in autoimmune diseases has not been investigated.

Mouse DNCD3 splenic cells can protect against lethal GVDH in a non-MHC restricted manner [6], [7]. However, the function of DNCD3 splenic cells in healthy human subjects, where they co-exist as a mixture of naïve MHC-restricted and antigen-experienced cells, is still unknown [2]. A regulatory mechanism of DNCD3 splenic cells in mice was described to rely on Fas-FasL mediated cytolysis of CD4 and CD8 T-cells, since the defective Fas-FasL signaling in *lpr/lpr* mice displaying massive lymphoproliferation was associated with a lack of DNCD3 cytolytic activity [8].

It has been postulated that the DNCD3 splenic cells originate in thymus by escaping negative selection followed by migration in periphery where they expand upon experiencing antigen [9]. Using TREC (signal-joint TRECs and DJβTRECs) markers of thymic differentiation, it has been shown that DNCD3 cells may in fact undergo thymic positive selection prior to migration in the periphery [10], [11]. Thymic positive selection of IL-10-secreting, regulatory DNCD3 cells may also occur by re-differentiation of CD3^+^4^+^8^+^ double positive thymocytes that interact with high-affinity ligands expressed by thymic epithelial cells [12]. Other studies claimed an extra-thymic pathway for DNCD3 cell development in liver [13]–[15], bone marrow [16], [17], or in periphery by a mechanism of MHC class II-acquirement called trogocytosis [16], [18]. Although the DNCD3 splenic cells appear to be non-functional *in vitro*, antigen stimulation *in vivo* in the presence of bystander IL-2 secretion by activated CD4^+^ T-cells leads to their rapid expansion in periphery [19] and IL-10 secretion [12].

Peripheral DNCD3 cells can restrict the function of T-cells specific for allo- and xeno-antigens [20]–[23]. These cells can also restrict the immune response to infectious agents [13], [24] and suppress the growth of tumor cells [25]. In contrast, the blood circulating DNCD3 cells from SLE patients may exert inflammatory effects [26]. At present, there is little information about the role of DNCD3 peripheral cells in autoimmunity, particularly in type-1 diabetes (T1D), a disease characterized by the reduction of insulin production subsequent to destruction of pancreatic β-cells by a polyclonal population of *self*-reactive T-cells [27], [28]. The most appropriate model for human T1D is the NOD mouse that develops the disease spontaneously and displays a similar pathology to humans [29]. Like in humans, the autoimmune diabetes in NOD mice is mediated by a polyclonal population of T-cells reactive to a variety of *self-*peptides [30]. Besides the MHC and non-MHC genetic factors [31], the autoimmune diabetes in humans and NOD mice is associated with a large number of qualitative and quantitative defects in the T-cell compartment [32], [33].

Herein, we have analyzed for the first time the phenotype and function of DNCD3 splenic cells from young NOD mice. These cells displayed a TCR Vβ13-biased usage apart from that of canonical NKT cells, and lacked expression of the Foxp3 marker of naturally-occurring CD4^+^CD25^high^ T-reg cells. DNCD3 splenic cells were able to proliferate in the spleen and pancreas of NOD/Scid immunodeficient mice and pre-diabetic NOD mice, and to differentiate into CD4^+^8^+^ double positive T-cells and IL-10-secreting CD4^+^T_R_-1 like cells upon stimulation in Th2-like environment. Most importantly, the NOD DNCD3 splenic cells from young NOD mice induced long-lasting protection against diabetes transfer into NOD/Scid mice.

## Methods

### Ethics statement

Mice were purchased from Jackson Laboratories (Bar Harbor, ME) and housed in pathogen-free conditions at USUHS/LAM facility. Experiments and care/welfare were in agreement with federal regulations and an approved protocol by the USUHS/IACUC committee.

### Mice

NOD/Lt, NON.NOD *H-2^g7^*, and NOD.CB17-PrkdcSCID/J immune deficient mice (NOD/Scid mice) were purchased from Jackson Laboratories (Bar Harbor, ME). The NON.NOD mice are congenic for the NOD *H-2^g7^* haplotype, and they were used as diabetes-resistant, control mice.

### Cell isolation

Thymic cells, splenic cells, and pancreatic-infiltrating lymphocytes were obtained from a pool of mice, or in some experiments from individual mice. Pancreatic infiltrating lymphocytes were isolated using either the collagenase or tissue douncing method. For the collagenase method, 10^7^ cells were treated with 4 mg/ml proteinase-free collagenase (Sigma-Aldrich, # C6079) for 20 min at 37°C in saline solution pH 7.5 under gentle agitation followed by neutralization of collagenase with an equal volume of complete RPMI media. Cells were centrifuged at 800×*g*, re-suspended in saline solution containing 1% BSA, and passed through a 100 µm filter mesh before analysis. For the tissue douncing method, the pancreas was gently dounced, passed through a 100 µm filter mesh (BD PharMingen, San Diego, CA), and centrifuged at 200×*g* in RPMI medium supplemented with 10% heat-inactivated fetal calf serum to remove islets and cell debris followed by centrifugation at 800×g. Negatively-sorted CD4 T-cells were obtained at higher than 90% purity according to FACS analysis by cell passage through mouse CD4 subset column kit # MCD4C according to the manufacturer's instructions (R&D Systems, Minneapolis, MN). DNCD3 T-cells were isolated either by depletion of CD4 and CD8 T-cells using tandem CD4 and CD8 mouse column kits (# MCD4C and #MCD8C 1000, R&D Systems), or by FACSAria cell sorter (BD, San Jose, CA) at 98% purity (Figure S1). For purification of DN, DP, and SP4 T-cell subsets, single cell suspensions were triple stained with CD3 Ab-FITC, CD4 Ab-PE, and CD8 Ab-PerCP conjugates (BD PharMingen, CA) and then FACS-sorted in 3 simultaneous windows in a FACSAria instrument. In some experiments, the TCRγδ/NK cell depletion of FACS-sorted DNCD3 splenocytes was carried out by incubation of cells with 2 µg/10^6^ cells of anti-mouse TCRγδ Ab-PE (clone #GL3, BD PharMingen, San Jose, CA) and 2 µg/10^6^ cells of anti-asialo-GM1 Ab-PE conjugates (clone #SH34, ATCC) followed by incubation with anti-PE Abs coupled to magnetic beads and passage on MACS paramagnetic columns according to the manufacturer's instructions (Miltenyi Biotech Inc., Auburn, CA).

### Cell cycle divisions

FACS-sorted DNCD3 splenic cells (10^6^ cells) were incubated on ice for 30 min in RPMI 1640 medium with 2.5 µg carboxyfluorescein succinimidyl ester (CFSE, Sigma-Aldrich, St. Louis, MO). The reaction was stopped by washing the cells for 10 min at 800×g and 4°C with RPMI medium supplemented with 10% FCS, followed by one wash in phosphate buffer saline (PBS) prior to infusion in NOD/Scid mice. CSFE labeling of cells *in vivo* was carried out according to our protocol [34]. Briefly, young NOD littermates of various ages were injected intraperitoneally (i.p.) with 0.1 mg CFSE (Sigma Chemicals, Inc, New Jersey, NJ) per gram of body weight. Seven days post-CFSE injection the total spleen cells were harvested and stained with various Ab-dye conjugates (BD PharMingen, San Jose, CA). Cell cycle divisions of the CFSE-labeled cells were detected in FACS-gated cell populations based on CFSE dilution factor using a LSR II instrument and WINlist analysis software 3D 5.0. (BD Biosciences).

### Single-Cell Flow Cytometry

Single-cell suspensions of thymocytes, splenocytes, or pancreatic infiltrating lymphocytes were phenotyped by 4-color staining in FACS using various Ab-dye conjugates specific for T-cell surface antigens (BD PharMingen). Differences in the mean fluorescence intensity (MFI) due to the cell size and signal-to-noise autofluorescence were compensated using the WINlist analysis software (Verity Software, Topsham, ME) during the data acquisition in a LSR II Becton Dickinson instrument.

### Cell cultures and cytokines assays

Single-cell suspensions (10^6^ cells) from spleens pooled from each group of mice were cultured in 96-well plates for 1, 3, or 5 days in RPMI medium supplemented with 10% FCS in the presence or absence of 2.5 µg/ml of each CD3ε Ab (ATCC, #2C11) and CD28 Ab (ATCC, #7D4). In some experiments, negatively-sorted DNCD3 splenic cells on mouse CD4/CD8 tandem columns cells were cultured for 5 days in Th1 conditioned medium (10% FCS RPMI medium supplemented with 10 U/ml rIL-12, 10 µg/ml anti-IL-4 mAb, and 2.5 µg/ml CD3/CD28 Abs) or in Th2 conditioned medium (10 µg/ml anti-IFN-γ mAbs and 2.5 µg/ml CD3/CD28 Abs). The cytokine secretion in cell culture supernatants was measured by ELISA kits using the manufacturer's protocol (BioSource International, Camarillo, CA). For detection of Foxp3 gene expression, the FACS-sorted DNCD3 splenic cells were stimulated for 1 day with CD3 and CD28 mAbs in T-reg conditioned medium containing TGF-β 100 ng/10^6^ cells).

### RT-PCR

RNA was isolated using the PureLink RNA purification system (Invitrogen, Carlsbad, CA) from various cell subsets, i.e., negatively-sorted CD4 splenic T-cells, or FACS-sorted DNCD3 splenic cells or DN, DP, and CD4 single positive thymocytes (SP4) stimulated or not under Th1, Th2, or T-reg conditions. Total RNA (4 ng) was used to prepare first-strand cDNA (One Step RT-PCR kit, Qiagen, Valencia, CA) following the manufacturer's protocol. The specific primer pairs for Foxp3 were: (forward) 5′ CAGCTGCCTACAGTGCCCCTAG, and (reverse) 5′ CATTTGCCAGCAGTGGGTAG; pre-TCRα (forward) 5′CTGCAACTGGGTCATGCTTC3′ and (reverse) 5′TCAGAGGGGTGGGTAAGATC3′), and for RAG2 (forward) 5′TGCTAACTTGTATAGAATAAGAGT and (reverse) 5′TCTTCTGTTGATGTCTGACTGT T). The specific primers for GATA3, NF-ATc, STAT4, STAT6, T-bet, IL-10, β-actin, and GAPDH were purchased from Applied Biosystems. PCR products (25 µL) were electrophoresed in ethidium bromide/1.5% agarose gels and the amplicon bands were visualized in a KODAK imaging system.

### Adoptive cell transfer protocols

Groups of 1–2 month-old NOD/Scid female mice were infused i.p. with either diabetogenic spleen cells from hyperglycemic NOD mice (control diabetes), or FACS-sorted DNCD3 splenic cells, or co-infused with FACS-sorted DNCD3 splenic cells and diabetogenic cells, or TCRγδ /NK-depleted, FACS-sorted DNCD3 splenic cells from young NOD or NON.NOD (control) mice. In some experiments, infusion of DNCD3 splenic cells was followed either by i.v. injection of 4 doses of 100 µg/mouse of neutralizing IL-10 Ab (clone #JES-2AS, ATCC) prior to the co-infusion of diabetogenic cells, or by i.p. injection of CFSE as described. The DNCD3 cell preparations were co-infused either at the same time with diabetogenic splenocytes or 1 month prior to infusion of diabetogenic splenocytes. Adoptively cell transferred NOD/Scid mice were tested for blood glucose levels on a weekly-basis via optical bleeding using the Ascensia CONTOUR glucose strips (Bayer, City, IN). Mice were considered diabetic after two consecutive glucose readings greater than 200 mg/dL. Glucose tolerance tests in some groups of adoptively cell transferred NOD/Scid mice were carried out by i.p. injection of 60 mg glucose in saline per mouse followed by glycemia readings at various time intervals.

### Immunohistochemistry

Paraffin-embedded sections of pancreata were stained with hematoxylin-eosin to identify infiltrating lymphocytes. The extent of insulin secretion by pancreatic β-cells was estimated in adjacent pancreatic sections by staining with a rabbit anti-insulin Ab (1∶200 dilution) revealed by a goat anti-rabbit IgG-horseradish peroxidase (HRP)-conjugate (Santa Cruz Biotech, Santa Cruz, CA).

### Statistic analysis

Significance of survival and diabetes incidence in NOD/Scid mice receiving various cell preparations was compared with that of control groups (diabetogenic T-cells alone) by the Kaplan-Meier test, and the significance of intra-group individual variations was determined by Kruskal-Wallis non parametric variance test. The significance (*p*≤0.005) of individual differences in frequency of DNCD3 thymocytes and splenocytes from female and male NOD littermates of different ages was estimated by the *paired-*Student's *t*-test.

## Results

### Young NOD mice display high frequency of DNCD3 splenic cells

**Figure 1:**
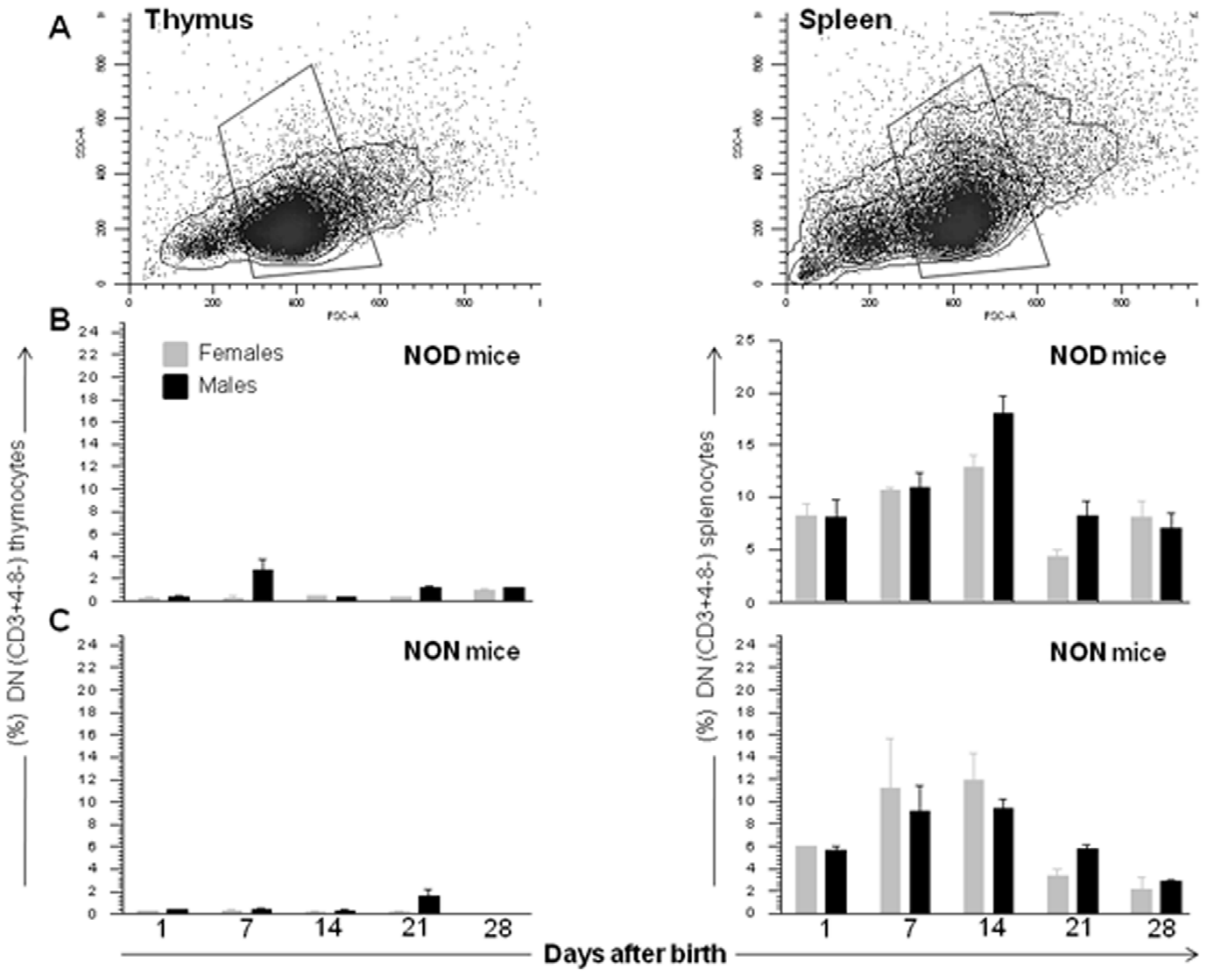
Frequency of NOD DNCD3 thymic and splenic cells. Total thymocytes and splenocytes from individual NOD and NON.NOD control males and females were harvested at various time-points after birth, stained with CD3-PE, CD4-PE-Cy7, and CD8-PerCP mAb-conjugates, and the frequency of CD3^+^4^−^8^−^ (DNCD3) cells was measured by FACS. *Panel A*, cells gated based on the forward and light scatter to identify viable populations. *Panel B*, kinetics of thymic *vs*. splenic DNCD3 cell development in newborn NOD mice. *Panel C*, kinetics of thymic *vs*. splenic DNCD3 cell development in newborn NON.NOD control mice. Shown are the individual variations (mean ± SD) in each age group.

Previous studies showed no significant differences in the size of peripheral T-cell compartment between NOD and other mouse strains like BALB/c, B6, and CBA mice [35]. We measured the DNCD3 cell population size in the thymus and spleen of age-matched NOD and NON.NOD (control) males and females (n = 18 mice per group) from 3 different litters by FACS at several time-points after birth (Figure 1A). The number of DNCD3 cells was lower in the thymus than spleen when analyzed between 2 to 28 days after birth in both male and female NOD mice (Figure 1B). The highest number of DNCD3 thymocytes in newborn NOD mice was detected 7 days after birth in males, but not female littermates.

The spleen of both male and female NOD mice showed a gradual increase in the number of DNCD3 cells with a peak at day 14 after birth (10–22% of the entire CD3^+^ cell compartment) (Figure 1C), which was followed by a decline up to 1–3% of the entire CD3^+^ cell population some 28 days after birth. This was consistent with previous data showing a relative low number of DNCD3 peripheral cells in NOD mice older than 5 weeks [36]. These data indicated that regardless the gender, the number of DNCD3 splenic cells in NOD mice was relatively high during the postnatal period, and sharply declined in the adulthood.

### NOD DNCD3 splenocytes are not diabetogenic, but rather tolerogenic

Several studies indicated that peripheral DNCD3 cells are endowed with regulatory functions. Since the number of DNCD3 splenic cells was relatively high in young NOD mice, we first tested their function *in vivo*. To our knowledge, a single report described a regulatory function of DNCD3 splenic cells in a double transgenic mouse model for T1D [37]. In this model, the mice express a TCR specific for LCMV gp33–41 peptide and the LCMV gp protein expressed in β-pancreatic cells. Diabetes in these mice does not develop spontaneously, and it could be induced only by parenteral co-administration of gp33 peptide and a CD40 agonist antibody. Enrichment of DNCD3 splenic cell number in these mice by adoptive transfer was associated with a delay in the onset of diabetes. However, the transgenic mouse models for autoimmunity have two major caveats. First, the autoimmunity is limited to a single antigen in contrast to the polyclonal antigen-specificity of T-cells in humans and NOD mice. Second, the transgenic mouse models display aberrant expression of peptide-specific TCRα and β transgenes, which bypasses the thymic negative selection and consequently lead to the escape of an unusual large number of autoreactive T-cells in periphery [38].

**Figure 2:**
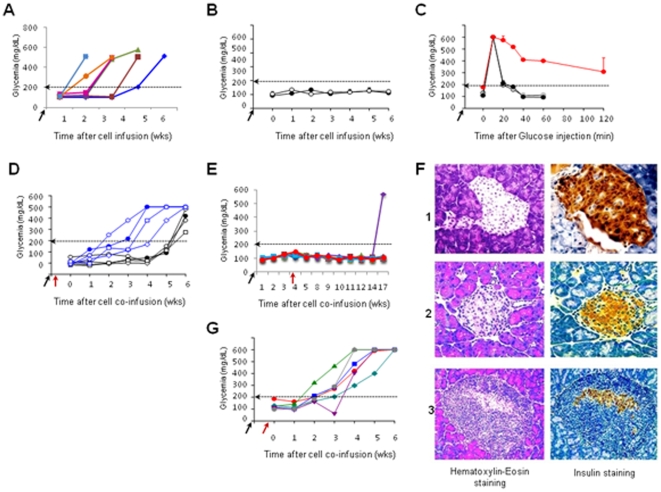
NOD DNCD3 protection against diabetes transfer in NOD/Scid mice. *Panel A*, diabetogenic splenocytes (5×10^5^ cells) from a pool of 4–5 month-old diabetic (hyperglycemic) NOD mice were injected i.p. into 4 week-old NOD/Scid female mice (n = 6, control diabetes group) and glycemia was measured on a weekly-basis. Dotted horizontal line at 200 mg/dL indicates the upper limit of euglycemia as previously established in a cohort of 12 non-treated, 4 week-old NOD/Scid females. Colored symbols refer to individual mice. Arrow on the X-axis indicates the time of cell infusion. *Panel B*, FACS-sorted DNCD3 splenic cells (5×10^5^ cells) from a pool of 14 day-old NOD (dark symbols) and NON.NOD females (light symbols) (n = 20 donors per group) were infused i.p into 4 week-old NOD/Scid females (n = 6 recipients per group) and glycemia was monitored on a weekly-basis. Shown are the glycemia values (mean ± SD) in each group of NOD/Scid recipients. Arrow on the X-axis indicates the time of cell infusion. *Panel C*, glucose tolerance test carried out in NOD/Scid mice 2 months after infusion of DNCD3 splenocytes from 14 day-old NOD mice. Shown are two representative NOD/Scid mice infused with diabetogenic splenic cells from a hyperglycemic NOD mouse and tested 2 weeks post-cell infusion (diabetes control, red symbols); a representative NOD/Scid mouse infused with DNCD3 splenocytes from 14 day-old NOD mice (grey symbols), and a representative NOD/Scid mouse infused with DNCD3 splenocytes from 14 day-old NON.NOD mice (white symbols). Arrow on the X axis indicates the time of glucose i.p. injection (60 mg/mouse). *Panel D*, FACS-sorted DNCD3 splenic cells (5×10^5^ cells) from 14 day-old NOD or NON.NOD females (pool of 20 mice per group) were co-infused i.p. in the same day with diabetogenic splenocytes (5×10^5^ cells) from diabetic NOD females into 4 week-old NOD/Scid females (n = 6 recipients per group). Shown are the glycemia values in individual NOD/Scid recipients of DNCD3 splenocytes from NOD mice (dark symbols) or NON.NOD control mice (blue symbols). Arrows (dark and red) indicate the same time of cell co-infusion. *Panel E*, FACS-sorted DNCD3 splenic cells (5×10^5^ cells) from 14 day-old NOD females (pool of 20 mice) were infused i.p. and 1 month later co-infused i.p. with diabetogenic splenocytes (5×10^5^ cells) from diabetic NOD females into 4 week-old NOD/Scid females (n = 6 recipients/group). Shown are the glycemia values in individual NOD/Scid recipients. The time-points of cell infusion with DNCD3 splenocytes (dark arrow) and co-infusion of diabetogenic cells (red arrow) are indicated. A single NOD/Scid recipient became hyperglycemic 10 weeks after co-transfer, whereas the other 5 NOD/Scid mice remained euglycemic (p = 0.002 for the group of protected NOD/Scid mice by NOD DNCD3 splenocytes as compared with non-protected NOD/Scid mice by NON.NOD DNCD3 splenocytes). *Panel F*, hematoxylin-eosin staining of paraffin-embedded sections of the pancreas for a representative euglycemic, non-injected NOD/Scid mouse (row 1, left panel); an euglycemic NOD/Scid mouse 13 weeks after co-transfer of NOD DNCD3 splenocytes with diabetogenic splenocytes as in panel E (row 2, left panel); and the only hyperglycemic NOD/Scid mouse 13 weeks after cell co-transfer of NOD DNCD3 splenocytes with diabetogenic splenocytes as in panel E (row 3, left panel). Shown is the staining of adjacent pancreatic cross-sections from the same mice with a rabbit anti-mouse insulin Ab-HRP conjugate (rows 1–3, right panels). *Panel G*, FACS-sorted DNCD3 splenic cells (5×10^5^ cells) from 14 day-old NON.NOD females (pool of 20 mice) were infused i.p. into 4 week-old NOD/Scid females (n = 6 recipients per group) and 1 month later co-infused i.p. with diabetogenic splenocytes (5×10^5^ cells) from diabetic NOD females. Shown are the glycemia values in individual NOD/Scid recipients. The time-points of cell infusion with DNCD3 splenocytes (dark arrow) followed 1 month later by co-infusion of diabetogenic cells (red arrow) are indicated. All NOD/Scid recipients became hyperglycemic within 3 to 4 weeks as the diabetes control group did (see panel A) (p≤0.001 for two combining experiments). Each panel represents one of two representative experiments for which the significance of diabetes incidence was p ≤0.005.

Herein, we compared the function of DNCD3 splenic cells from young NOD *H-2^g7^*and age-matched NON.NOD *H-2^g7^* (diabetes resistant) mice in adoptive cell transfer experiments using NOD/Scid recipients. Naïve (non-manipulated) NOD/Scid mice are normoglycemic and their pancreatic β-islets have robust insulin secretion. NOD/Scid mice infused with diabetogenic splenocytes (5×10^5^ cells/mouse) isolated from 4–5 month-old hyperglycemic NOD mice (diabetes-control group) developed hyperglycemia and pancreatic insulitis within 4–5 weeks (Figure 2A). None of the NOD/Scid recipients infused with DNCD3 splenocytes (5×10^5^ cells/mouse) isolated from 14 day-old NOD or NON.NOD mice developed hyperglycemia (Figure 2B) or pancreatic insulitis (not shown), and they all showed a normal glucose tolerance test 2 months after the cell transfer (Figure 2C). This clearly showed a lack of diabetogenicity of DNCD3 splenic cells from young NOD mice when parked into NOD/Scid immunodeficient mice.

We next tested the tolerogenicity of NOD *vs*. NON.NOD DNCD3 splenocytes in the NOD/Scid system. Groups of NOD/Scid mice received DNCD3 splenocytes (5×10^5^ cells/mouse) from a pool of 14 day-old NOD or NON.NOD control females at the same time with diabetogenic splenocytes (5×10^5^ cells/mouse) from 4–5 month-old hyperglycemic NOD mice. All the NOD/Scid recipients in these groups developed hyperglycemia, although those co-infused with NOD DNCD3 splenocytes and diabetogenic cells, but not those infused with NON.NOD DNCD3 splenocytes and diabetogenic cells showed a 2 to 3-week delay in hyperglycemia onset (Figure 2D). At first, the NOD DNCD3 splenic cells showed a tolerogenic effect that was insufficient to abrogate the onset of hyperglycemia in NOD/Scid mice co-infused at the same time-point with diabetogenic splenocytes. In contrast, five of six NOD/Scid mice in one group, and all five mice in another group receiving the same number of DNCD3 splenocytes (5×10^5^ cells/mouse) from 14 day-old NOD females 1 month prior to co-infusion of diabetogenic splenocytes (5×10^5^ cells/mouse) remained normoglycemic for 13 weeks. A single mouse in these two groups developed late hyperglycemia (10 weeks) after co-infusion of diabetogenic cells (Figure 2E). Protected NOD/Scid recipients showed normal morphology of the pancreatic islets and strong β-cell secretion of insulin 13 weeks after co-infusion with diabetogenic cells (Figure 2F, middle rows). The only mouse in these groups that developed late hyperglycemia showed pancreatic insulitis and reduction in β-islet insulin secretion (Figure 2F, bottom rows). Hyperglycemia and pancreatic insulitis were detected in all NOD/Scid recipients (n = 6) of NON.NOD DNCD3 splenic cells (5×10^5^ cells/mouse) infused 1 month prior to co-infusion of diabetogenic splenocytes (5×10^5^ cells/mouse) (Figure 2G).

There is a body of evidence demonstrating that the number and function of NK and NKT cells in the NOD mice are deficient. Adoptive transfer of NK cells in some mouse models of diabetes can prevent/delay the onset of disease [39]–[41]. Also, the function of CD3^+^(4^−^8^−^) double negative TCRγδ^+^ cells in autoimmune diabetes remains poorly defined. Herein, we tested whether the anti-diabetogenic function of NOD DNCD3 splenic cells could be attributed to some extent to a small number (1–2%) of contaminating CD3^+^(4^−^8^−^) double negative TCRγδ^+^ cells in the preparations of FACS-sorted DNCD3 splenic cells. For this, we injected a group of NOD/Scid mice with FACS-sorted DNCD3 splenocytes (5×10^5^ cells/mouse) isolated from 14 day-old NOD females followed by CFSE *in vivo* labeling, as described. Seven days later, the CFSE dilution factor in CD4^−^8^−^-gated TCRγδ cells indicated a lack of cell division (Figure S2). Furthermore, a separate group of NOD/Scid mice was infused with TCRγδ/NK-depleted, FACS-sorted DNCD3 splenocytes (5×10^5^ cells/mouse) isolated from 14 day-old NOD females followed by co-infusion of diabetogenic cells (5×10^5^ cells/mouse) 1 month later. All the recipient mice in this group remained normoglycemic until the present time (8 weeks after the cell co-transfer, data not shown). These data clearly ruled out the possibility that a small number of contaminating TCRγδ^+^ cells or NK cells in the FACS-sorted DNCD3 cell preparations could expand and contribute to the DNCD3 anti-diabetogenicity in the NOD/Scid system.

Together, these results demonstrated that the DNCD3 splenic cells from young NOD mice are not diabetogenic when parked in immunodeficient mice, but rather exerted a tolerogenic (anti-diabetogenic) effect. A lag period was required for the NOD DNCD3 splenic cells to develop a strong anti-diabetogenic effect in the NOD/Scid mice. In contrast, the NON.NOD DNCD3 splenic cells exerted neither diabetogenic nor anti-diabetogenic effects in NOD/Scid mice.

### NOD DNCD3 splenocytes proliferate and differentiate *in vivo*

**Figure 3:**
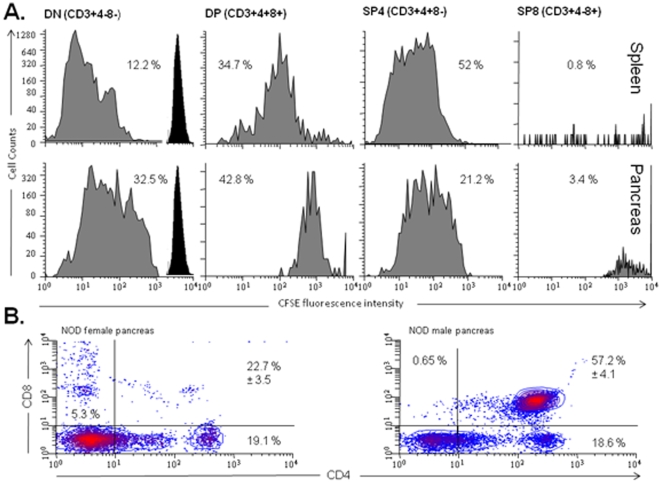
*In Vivo* proliferation and differentiation of NOD DNCD3 splenic cells. FACS-sorted DNCD3 splenic cells from a pool (n = 20) of 14 day-old NOD females were labeled ex-vivo with CFSE and injected i.p. into three NOD/Scid mice (5×10^6^ cells per mouse). Seven days post-infusion, the spleen and pancreas of recipient mice were harvested, the cells were isolated by douncing, and single-cell suspensions were pooled and stained with CD4-PE-Cy7 and CD8-PerCP mAb-dye conjugates. Shown are the cycles of cell division of CD3^+^4^−^8^−^ (DN), CD3^+^4^+^8^+^ (DP), CD3^+^4^+^8^−^ (SP4), and CD3^+^4^−^8^+^ (SP8) cell populations from the spleen (*panel A, upper histograms*) and pancreas (*panel A, lower histograms*) of the NOD/Scid infused with CFSE-labeled DNCD3 splenocytes. The percent in each cell subset (grey histograms) and CFSE-cell intensity (dark peaks, cycle “0” of division) are indicated. *Panel B*, pancreatic infiltrating lymphocytes from 4 month-old NOD females (*left histogram*) and males (*right histogram*) (n = 8 mice/group) were isolated and stained for CD3, CD4 and CD8 markers as described. Shown are the mean values (%) of DN, DP, SP4 and SP8-like cell populations for two representative experiments ± SD values for individual male and female NOD mice.

The ability of DNCD3 splenic cells to proliferate in some mouse strains has been previously described [21], [42]. Since the NOD DNCD3 splenic cells required a lag period (1 month) of being parked in NOD/Scid mice in order to exert a strong anti-diabetogenic effect, we investigated the fate of these cells in NOD/Scid mice. For this, a group of NOD/Scid mice was infused with *in vitro* CFSE-labeled DNCD3 splenic cells (5×10^6^ cells/mouse) that were FACS-sorted from a pool of 14 day-old NOD female mice, and 7 days later the CFSE^+^ cells were harvested from the spleen and pancreas of recipient mice and the cell cycle division was measured by FACS in the CD3-gated population. Some 12% and respectively 32% of CFSE-labeled DNCD3 cells advanced to 5–6 cycles of cell division in the spleen (Figure 3A, upper panels) and pancreas (Figure 3A, lower panels). Some 35% and respectively 42% of the CFSE^+^-proliferating cells expressed both the CD4 and CD8 markers (CD3^+^(4^+^8^+^) double positive T-cells), and 52% and respectively 21% expressed only the CD4 marker (CD3^+^4^+^8^−^ single positive T-cells) in the spleen and pancreas. A few CFSE^+^ labeled CD3^+^(4^−^8^+^) single positive T-cells were detected in the spleen (0.8%) and pancreas (3.4%) of NOD/Scid recipients. These results indicated a process of DNCD3 cell differentiation into more mature T-cells after being parked in the NOD/Scid immunodeficient mice. The analysis of DNCD3 splenic cells in CFSE-labeled, non-manipulated NOD mice at various time-points after birth also showed a gradual increase in their rate of proliferation with the peak at day 14 after birth (Figure S3), which was consistent with our previous data showing a high percent of DNCD3 cells found in the spleen of young NOD mice 14 days after birth.

Furthermore, the CD3^+^ pancreatic infiltrating lymphocytes from CFSE-injected, 4 month-old pre-diabetic NOD mice also showed the presence of CD3^+^(4^+^8^+^) double positive T-cells with a 3-times higher frequency in males than females (57% *vs*. 22%) (Figure 3B). The pancreas of CFSE-injected, 4 month-old pre-diabetic NOD females showed a significantly higher number of CD3^+^(4^−^8^+^) single positive T-cells (5.3%) as compared with their male littermates (0.6%). Identification of CD3^+^(4^+^8^+^) double positive T-cells by FACS in single-cell suspensions of pancreatic infiltrating lymphocytes from pre-diabetic NOD mice was possible only by douncing the pancreas instead of using the collagenase method of tissue digestion. We found that two different preparations of collagenase had drastically altered the phenotype of T-cells, since shortly after digestion of thymus with collagenase the CD4^+^8^+^ double positive thymocytes lost the surface expression of CD4 and CD8 antigens to a large extent (Figure S4), most probably due to contaminating proteases in collagenase preparations. The presence of double positive T-cells in the pancreas of pre-diabetic mice suggested a process of cell differentiation similar to that observed in the NOD/Scid mice infused with NOD DNCD3 splenocytes.

**Figure 4:**
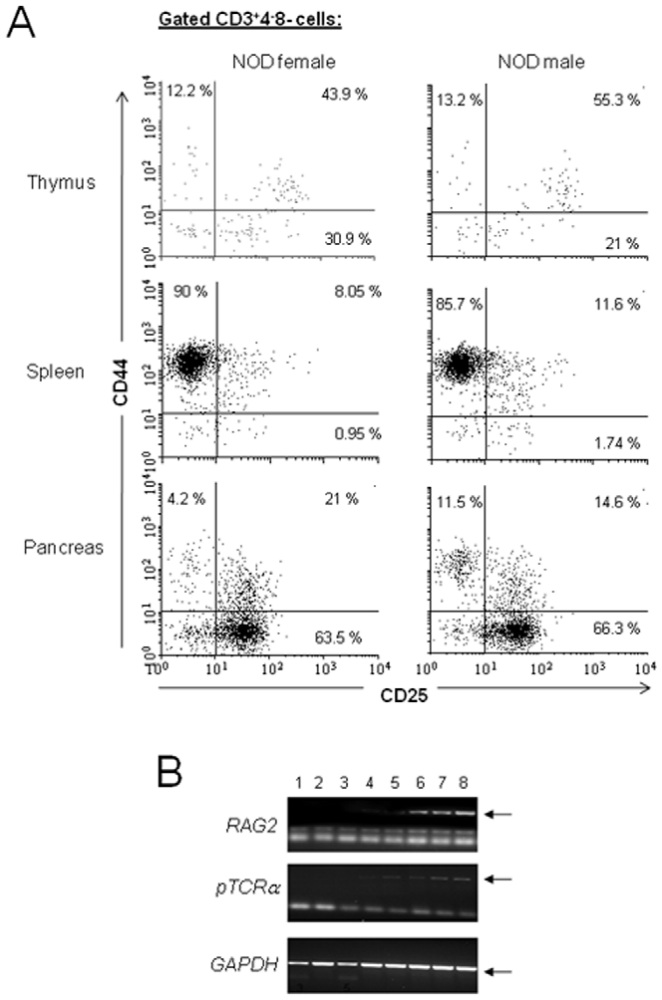
*In Vivo* detection of markers for NOD DNCD3 cell differentiation. *Panel A*, Single-cell suspensions isolated by tissue douncing of total thymocytes and splenocytes from 14 day-old individual NOD females and males from two different litters (n = 5 mice per gender), as well as pancreatic lymphocyte infiltrates from 4–5 month-old individual pre-diabetic NOD females were stained with CD3-Alexa 700, CD4-PE-Cy7, CD8-PerCP, CD25-APC, and CD44-FITC mAb-conjugates, and analyzed by FACS. Shown are the mean values (percent) of CD44 *vs.* CD25 expression on DNCD3 cells in each organ and gender ± SD. *Panel B*, patterns of RAG2 (upper panel) and pre-TCRα (middle panel) mRNA expression detected by RT-PCR in the FACS-sorted thymic, splenic and pancreatic infiltrating DNCD3, DP, and mature SP4 T-cell subsets from a pool of 4 to 5 month-old, pre-diabetic NOD female mice (n = 6), as described. *Lane 1*, SP4 pancreatic infiltrating cells; *lane 2*, SP4 splenocytes; *lane 3*, SP4 thymocytes; *lane 4*, DP pancreatic infiltrating cells; *lane 5*, DP thymocytes; *lane 6*, DNCD3 pancreatic infiltrating cells; *lane 7*, DNCD3 splenocytes; and *lane 8*, DN thymocytes. Lower panel shows the corresponding GAPDH mRNA amplicons analyzed in the upper and middle panels. Arrows indicate the presence of RAG2, pTCRα, and GAPDH amplicons.

A number of cellular and molecular events delineate T-cell differentiation in the thymus. Among these events, the expression pattern of CD44 *vs*. CD25 markers together with the expression patterns of RAG2 gene and pre-TCRα chain provide a quite accurate assessment of DN1-4 stages of differentiation in thymus. RAG2 gene is critical for transition of DN to DP stage of thymic differentiation, and its expression is gradually diminished in DP thymocytes and mature SP4 T-cells. At the same time, pTCRα gene expression is detected in the DN3-4 stage of thymic differentiation, and its expression diminishes to undetectable levels in DP stage and mature SP4 T-cells [43]–[45]. We first compared the pattern of CD25/CD44 expression in DNCD3 thymocytes and splenocytes to that of pancreatic infiltrating DNCD3 lymphocytes isolated by the tissue douncing method. The thymus contained 12–13% cells with a CD44^+^25^−^ (DN1) phenotype, 44–55% with a CD44^+^25^+^ (DN2) phenotype, 21–31% with a CD44^−^25^+^ (DN3) phenotype, and 11–13% with a CD44^−^25^−^ (DN4) phenotype (Figure 4). While the DN2 cells represented the major population in thymus (44–55%), most of DNCD3 splenocytes (90% in females and 86% in males) expressed a CD44^+^25^−^ phenotype, whereas most of pancreatic DNCD3 cells expressed a CD44^−^25^+^ phenotype. The pattern of RAG2 gene and pTCRα chain expression was also similar in the thymic, splenic, and pancreatic infiltrating DNCD3, DP, and mature SP4 T-cells isolated from 4–5 month-old pre-diabetic NOD females (Figure 4B). Similar results were obtained in pre-diabetic NOD males (data not shown). According to these results, and considering that CD3^+^(4^+^8^+^) double positive T-cells were almost undetectable in the spleen of 4–5 month-old pre-diabetic NOD mice (0.2–0.6%) while being present in the pancreatic infiltrating lymphocytes, it is not unlikely that the pancreatic infiltrating CD3^+^(4^+^8^+^) double positive T-cells derived from differentiation of pancreatic infiltrating DNCD3 cells.

**Figure 5:**
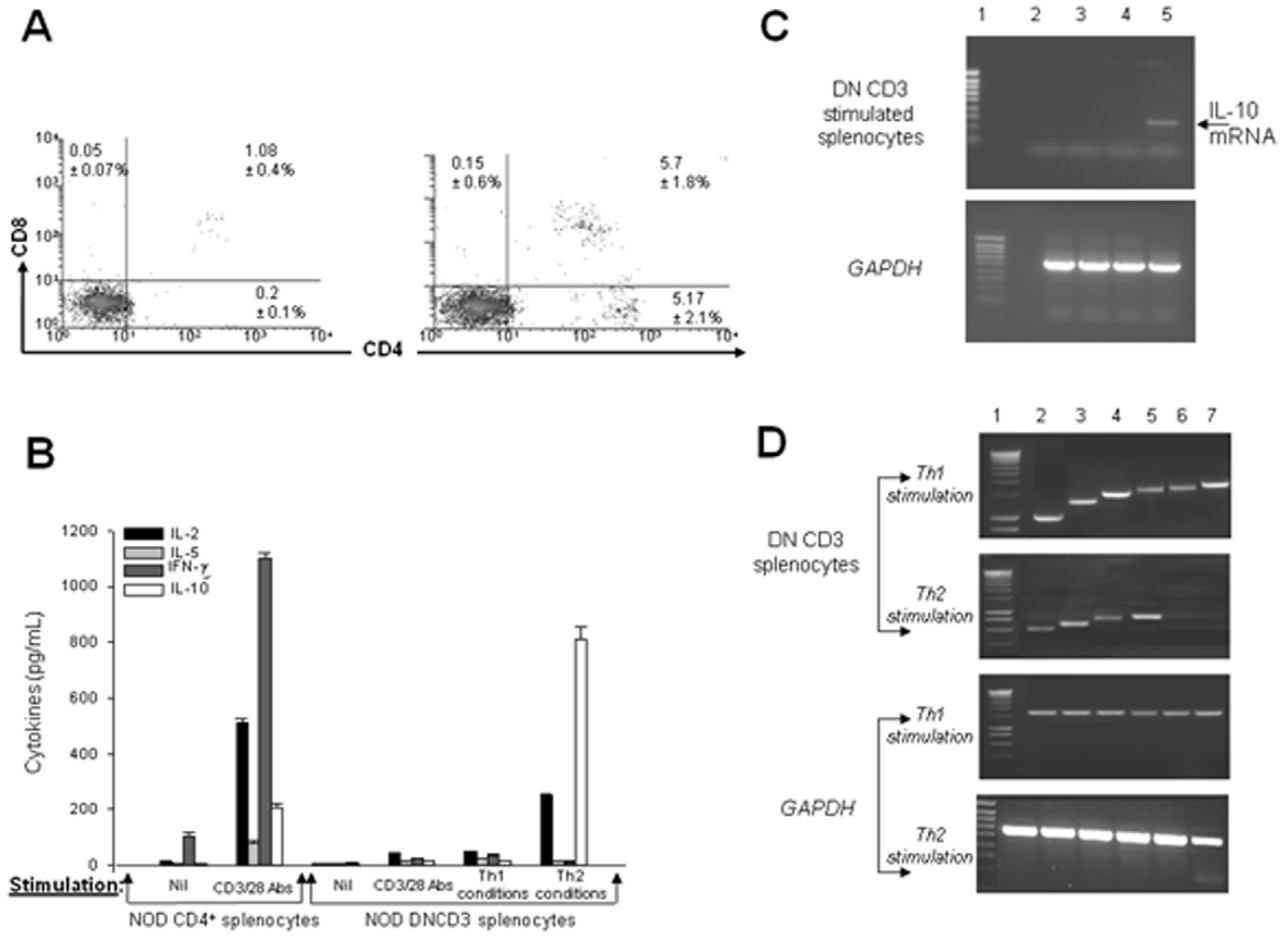
Cytokines and transcriptional events in NOD DNCD3 splenic cells. The DN splenic cells and mature, splenic CD4^+^ T-cells from a pool (n = 10) of 14 day-old female mice were negatively-selected on mouse CD4/CD8 tandem columns at 95% purity according to FACS analysis. Cells (1×10^6^) were stimulated or not for 1, 3, or 5 days with a mixture of soluble CD3/CD28 Abs (2.5 µg each), or with CD3/CD28 mAbs in Th1 or Th2 conditioned medium, as described. The one-day stimulation assay was used to measure the IL-2 secretion in the culture medium. *Panel A*, DN splenic cell cultures stimulated under Th1 (*left panel*) and Th2 (*right panel*) conditions, then stained 3 days later with CD4 Ab-APC and CD8 Ab-PerCP, and analyzed by FACS. Shown is the mean values (%) of CD4^−^8^+^ cytotoxic, CD4^+^8^+^ double positive, and CD4^+^8^−^ single positive T-cells from duplicate cultures ± SD. *Panel B*, mean values of cytokines measured in duplicate wells by ELISA in the same DNCD3 cell cultures (pg/mL ± SD) described in *panel A*. *Panel C*, mRNA extracted 3 days after stimulation of aliquot DNCD3 cell cultures like in *panels A&B*, and amplified in RT-PCR using specific primers for IL-10. In the upper panel, lane 1, molecular markers; lane 2, non-stimulated cells after 1 day of culturing in medium alone; lane 3, CD3/CD28 stimulated cultures; lane 4, cell cultures stimulated in Th1 conditioned medium; and lane 5, cell cultures stimulated in Th2 conditioned medium. Lower panel shows the corresponding GAPDH mRNA amplicons for the corresponding samples analyzed in the upper panel for IL-10 mRNA expression. *Panel D*, mRNA extracted 3 days after stimulation from aliquot DNCD3 cell cultures like in *panels A&B*, and amplified in RT-PCR using specific primers for the major Th1 and Th2 transcription factors (*lane 1*, molecular markers, *lane 2*, STAT6; *lane 3*, GATA-3; *lane 4*, cMAF; *lane 5*, NF-ATc; *lane 6*, STAT4, and *lane 7*, T-bet). Lower panel shows the corresponding GAPDH mRNA amplicons for each transcription factor. Each panel shows one of two representative experiments.

The differentiation potential of DNCD3 splenic cells was also observed upon *in vitro* Th2 stimulation, but not CD3/CD28 or Th1 stimulation. Thus, a 3-day stimulation of negatively-sorted DNCD3 splenic cells isolated from 14 day-old NOD mice in Th2 conditioned medium led to the appearance of CD3^+^(4^+^8^+^) double positive T-cells, CD3^+^(4^+^8^−^) single positive T-cells, and few CD3^+^(4^−^8^+^) single positive T-cells (>1%) (Figure 5A, right panel). Aliquots of these cell cultures showed predominant IL-10 secretion after 3 days of culturing (Figure 5B). Although the IL-10 mRNA expression was absent in non-stimulated NOD DNCD3 splenic cells, its expression was detected by RT-PCR after 3 days of Th2-stimulation, which coincided with the appearance of mature CD4 T-cells in the cell cultures (Figure 5C). In contrast to Th2-stimulated cultures, there were no double positive T-cells and single positive T-cells, nor cytokine secretion detected in DNCD3 splenic cells stimulated for 3 and 5 days in Th1 conditions or CD3/CD28 Abs alone (Figure 5A left panel, and 5B). After 3 days of Th1-stimulation, the DNCD3 cell cultures showed expression of both Th1 and Th2 transcriptional factors, whereas the Th2-stimulated cultures lacked expression of Th1 transcription factors T-bet and STAT4 (Figure 5D). These results clearly indicated a biased differentiation of DNCD3 splenic cells into a T_R_-1 like phenotype.

To test whether IL-10 secretion by DNCD3 differentiating splenocytes plays a role in protection against diabetes transfer in NOD/Scid mice, a group of NOD/Scid mice was first infused with TCRγδ/NK-depleted, FACS-sorted DNCD3 splenocytes (5×10^5^ cells/mouse) from 14 day-old NOD females followed by i.v. injection of 4 doses of IL-10 neutralizing Ab (100 µg/mouse every other week). On day 30 after cell infusion and anti-IL-10 treatment, the NOD/Scid recipients were co-infused with diabetogenic splenocytes (5×10^5^ cells/mouse) from a pool of hyperglycemic NOD mice. Under these conditions, all five recipient mice developed hyperglycemia within 4 weeks after co-infusion of diabetogenic cells (data not shown), indicating that IL-10 secreted by DNCD3 differentiating splenocytes played a critical role to their anti-diabetogenic effect.

Together, these results indicated that the DNCD3 splenic cells from young NOD mice can differentiate into more mature T-cells in a lymphopenic/immunodeficient environment like the NOD/Scid mouse, as well as in a well-established autoimmune environment like the pre-diabetic NOD mouse. The DNCD3 splenic cells were able to differentiate preferentially into IL-10-secreting T_R_-1 like (anti-diabetogenic) cells upon Th2 stimulation.

### NOD DNCD3 splenocytes express a unique phenotype

We next analyzed the expression level of several protein receptors involved in T-cell effector functions in relation to the anti-diabetogenicity of DNCD3 splenic cells from young NOD mice. The CD28 co-stimulatory molecule plays an important role in T-cell priming and differentiation, and its expression level is increased early after T-cell activation [46]. The frequency of NOD DNCD3 CD28^+^ splenocytes (78–86%) was similar to that of mature, splenic CD3^+^4^+^28^+^ T-cells (92–98%) (Table 1). However, the average number of DNCD3 CD28^+^ splenocytes in NOD females was constantly higher (86%) than in the male littermates (78%). On average, the expression level of CD28 on DNCD3 splenocytes measured by the mean fluorescence intensity (MFI) in single-cell FACS analysis was also similar to that of mature, splenic CD3^+^4^+^ T-cells (MFI = 192−199 *vs*. 180–187).

**Table 1:** Phenotypic markers expressed by the NOD DNCD3 splenic cells.

Cell markers	Mature CD3^+^4^+^ splenic T-cells (%/*average MFI*)		DNCD3 splenic cells (%/*average MFI*)	
	NOD males	NOD females	NOD males	NOD females
**CD28^+^**	**92**±**3.7/** *180*	**98**±**1.1/** *187*	**78**±**4.3/** *192*	**86**±**4.6/** *199*
**CD69^+^**	**10.3**±**1.2/** *114*	**11.4**±**1.4/** *125*	**19.2**±**2.7/** *131*	**16.8**±**3.2/** *127*
**CD25^low^**	**6.2**±**1.9/** *237*	**2.5**±**0.8/** *216*	**19.2**±**3.1/** *248*	**13.4**±**3.1/** *257*
**CD25^high^**	**2.2**±**0.9/** *1,020*	**1.9**±**1.1/**1,002	**0.08**±**0.02/** *1,021*	**0.09**±**0.01/** *1,015*
**iCTLA-4^+^**	**2.3**±**0.8/** *ND*	**2.8**±**1.2/** *ND*	**0.8**±**0.2/** *ND*	**0.45**±**0.3/** *ND*
**DX5^+^**	**2.17**±**1.1/** *ND*	**1.65**±**0.8/** *ND*	**0.4**±**0.3/** *ND*	**0.2**±**0.1/** *ND*
**CD1d/α-gal-ceramide TCR^+^**	**2.9**±**0.4/** *ND*	**1.7**±**0.6/** *ND*	**0.17**±**0.1/** *ND*	**0.1**±**0.08/** *ND*
**CD1d/α-unloaded TCR^+^**	**0.12**±**0.1/** *ND*	**0.11**±**0.1/** *ND*	**0.14**±**0.1/** *ND*	**0.12**±**0.09/** *ND*
**TCR Vβ**	**87**±**4.7/** *ND*	**85**±**3.8/** *ND*	**97.5**±**1.1/** *ND*	**95.8**±**3.2/** *ND*
**TCR Vβ**	**15**±**2.2/** *ND*	**13**±**2.5/** *ND*	**2.1**±**1.2/** *ND*	**3.9**±**1.1/** *ND*

Single-cell suspensions (10^6^ cells) isolated from the spleen of 14-day old individual NOD males and females (n = 5 per group) were co-stained with CD3-Alexa Fluor700, CD4-APC, CD8-PerCP Cy5.5 conjugates, and either one of the following Ab-PE conjugates specific for CD28, CD69, CD25, iCTLA-4, DX5, TCR Vβ or TCR Vδ antigens. Aliquot cells were co-stained with CD3-Alexa Fluor700, CD4-PE, CD8-PerCP, and a CD1d tetramer unloaded or loaded with α-galactosyl ceramide-APC conjugate. Shown are the mean values ± SD for the frequency (%) and single-cell density (MFI) of each phenotypic marker expressed by DNCD3 splenocytes and mature CD4 T-cells (control) as determined by FACS (*ND, not done).

A relative high number of DNCD3 splenocytes showed expression of the CD69 early marker of activation (16.8–19.2%) and CD25 late marker of activation (13.4–19.2%) as compared with mature, splenic CD3^+^4^+^8^−^ T-cells (CD69^+^, 10.3–11.4% and CD25^low^, 2.5–6.2%) (Table 1). This suggested that a significant fraction of DNCD3 splenocytes are under a continuous state of activation, which is in line with the hypothesis that DNCD3 cells can be activated in peripheral lymphoid organs [21], [42].

A high level of CD25 expression (CD25^high^) on CD4 T-cells is the hallmark of naturally-occurring CD4^+^Foxp3^+^ T-regulatory (T-reg) cells [47]. The number of NOD DNCD3 CD25^high^ splenic cells was very low (Table 1), and Foxp3 expression was undetectable in their quiescent state or after stimulation with CD3/CD28 Abs alone, or by culturing in a Th1, Th2, or T-reg conditioned medium containing TGF-β (Figure S5) The CTLA-4 co-receptor is also expressed lately on the activated T-cells, and it inhibits the TCR signaling through phosphatase-induced tyrosine de-phosphorylation of TCR ζ-chains [48]. High intracellular CTLA-4 expression (iCTLA-4) is increased in the CD4^+^Foxp3^+^ T-regulatory (T-reg) cells, and it synergizes with their suppressogenic function [49]. Co-expression of iCTLA-4 and Foxp3 in T-reg cells has been recently reported to suppress pancreatic islet autoimmunity [50.51]. In contrast to the CD28 expression on NOD DNCD3 splenocytes, the iCTLA-4 expression was hardly detected (0.45–0.8%). The lack of CD25^high^ expression and iCTLA-4/Foxp3 co-expression on the NOD DNCD3 splenic cells in the presence or absence of stimulation in T-reg conditioned medium indicated that these cells are phenotypically different than the conventional CD4^+^CD25^high^ Foxp3^+^ T-reg cells.

**Figure 6:**
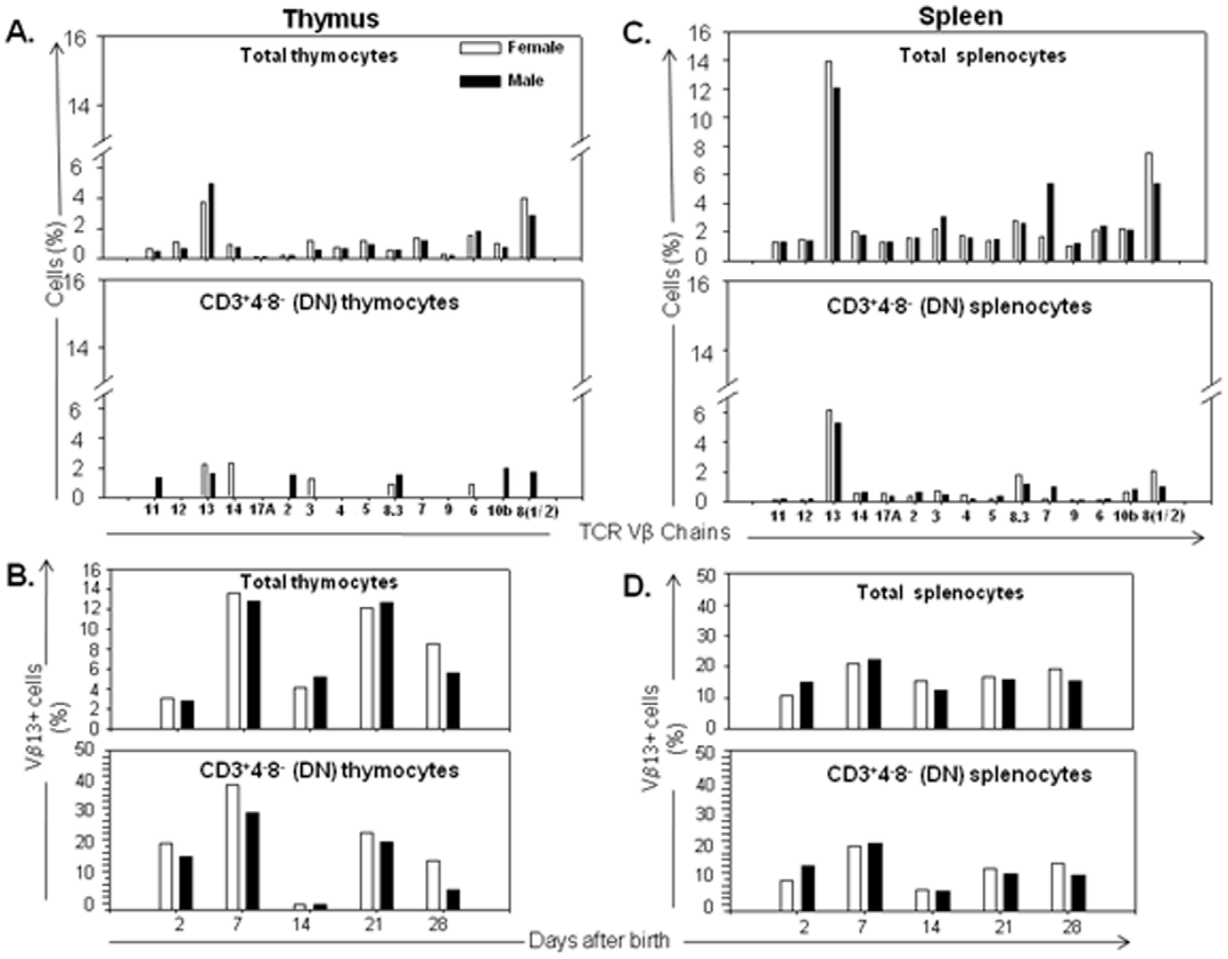
TCR Vβ repertoire of NOD DNCD3 splenic cells. Single-cell suspensions from the spleen and thymus of 14 day-old NOD female (n = 4) and male littermates (n = 3) were co-stained with monoclonal antibodies-FITC conjugates specific for several TCR Vβ-chains and with CD3-PE, CD4-PE-Cy7, and CD8-PerCP mAb-conjugates (Bioscience, Palo Alta, CA). *Panel A*, percentages of TCR Vβ families expressed on the cell surface of total thymocytes (*upper panel*) and DNCD3^+^ gated thymocytes (*lower panel*) as determined by FACS. *Panel B*, age-variation in the frequency of TCR Vβ13 on total thymocytes (*upper panel*) and DNCD3 thymocytes (*lower panel*) from NOD females (n = 3 per each group of age) and males (n = 3 per each group of age) as determined by FACS. *Panel C*, percentages of TCR Vβ families expressed on the cell surface of total splenocytes (*upper panel*) and DNCD3 splenocytes (*lower panel*) from the same mice analyzed by FACS as in panel A. *Panel D*, age-variation in the frequency of TCR Vβ13 on total splenocytes (*upper panel*) and DNCD3 splenocytes (*lower panel*) from the same NOD females (n = 3 per group of age) and males (n = 3 per group of age) as analyzed by FACS in panel B. Shown is the FACS analysis of a representative NOD female and male from two experiments (significance of Vβ13 family expression on DNCD3 cells between two separate experiments, p≤0.005).

NKT cells represent a small fraction of the DNCD3 peripheral pool in mice. However, the NK cell compartment is deficient in naïve (non manipulated) NOD mice [3], [4], [52]–[54], and infusion of a large number of competent NK cells from C57BL/6 mice in pre-diabetic NOD mice was shown to protect against diabetes onset [55]–[57]. We analyzed the expression of conventional NK biomarkers in NOD DNCD3 splenic cells. First, the expression of NK1.1 antigen on NOD DNCD3 splenic cells has been ruled out, since the NOD mouse strain like the C57BL/6 and SJL strains lacks the NK1.1 encoding allele. The number of DNCD3 splenocytes expressing the CD49b antigen recognized by DX5 mAb [58] was also insignificant (0.4±0.3%) as compared with mature CD3^+^4^+^ T-cells from the spleen of NOD mice (2.17±1.1%) (Table 1). Also, a CD1d tetramer loaded with α-galactosyl–ceramide surrogate of the glycosphingolipoidic moiety that is specifically recognized by NKT cells [59], was unable to stain the DNCD3 splenocytes from young NOD mice (0.17%±0.1% NKT cells), but it did stain mature, splenic NOD CD3^+^4^+^ T-cells (2.9±1.1% NKT cells) (Table 1). Since these results showed a lack of identity of NKT cells with the NOD DNCD3 splenocytes, we next analyzed the TCR repertoire of DNCD3 splenocytes in individual NOD mice from three different litters at various time-points after birth. NKT cells are known to display a biased expression of TCR Vα14: Vβ8.2 genes where Vα14 may also pair with Vβ7 or Vβ2 genes as found in the Vβ8 KO mice [60]. The TCR Vβ repertoire in DN thymocytes showed a predominant Vβ13 expression in both NOD males in females. Other Vβ families were well-spread, but lowly expressed, and with no gender differences in the DN thymocytes (Figure 6A). However, the expression of Vβ4, Vβ5, Vβ7, Vβ9, and Vβ14 gene families were undetectable 14 days after birth in both male and female littermates. The Vβ13 expression followed different kinetics during the postnatal period, with the highest expression 7 and 21 days after birth in NOD males and females (Figure 6B). The same distribution of Vβ families was found in the spleen of 14 day-old NOD females and males, although their expression level was slightly higher than in the DNCD3 splenic cells (Figure 6C). This was in agreement with the fact that Vβ is rearranged only in the DN3 stage of thymic differentiation [61]. Interestingly, only 2% of DNCD3 splenocytes expressed the Vβ8.2/3 gene products, whereas the Vβ13 gene product was predominantly expressed at day 14 after birth. The TCR Vβ13 followed a different kinetics of expression during the post-natal period in both genders (Figure 6D). These results clearly indicated that the TCRs on NOD DNCD3 splenocytes were encoded mostly by Vβ genes (95–97%) as compared with a small number encoded by Vδ genes (2–4%) (Table 1). Together, these data argue for a unique phenotype of NOD DNCD3 splenocytes apart from that of NKT cells. In conclusion, the DNCD3 splenic cells of young NOD mice showed the following predominant phenotype: CD3^+^ (CD4^−^CD8^−^) CD28^+^ CD69^+^ CD25^low^ Foxp3^−^ iCTA-4^−^ TCRαβ^+^.

## Discussion

T1D onset greatly depends on the balance between pancreatic *self*-reactive (diabetogenic) T-cells and T-reg compartment. T-reg cells are present in the peripheral lymphoid organs of NOD mice some 3 days after birth, but the adult NOD mice display quantitative and qualitative defects in the T-reg compartment [7] including the NK cells [50], [51]. Diabetes develops in NOD mice 12–14 wks after birth along with a loss in T-reg number and function and increase in the number of diabetogenic infiltrating T-cells in pancreas. Conceivably, the T-reg compartment may efficiently down-regulate the diabetogenic T-cell function in young, but not in adult NOD mice. The T-reg cell compartment comprises a heterogeneous population of cells, among which the recently described subset of CD3^+^(4^−^8^−^) double negative TCRαβ^+^ T-cells (DNCD3 cells) [62].

We found a high frequency of DNCD3 cells in the spleen of young NOD mice, and their number sharply declined in adulthood. DNCD3 splenic cells from young NOD mice were able to provide long-lasting protection (13 weeks) against diabetes transfer in NOD/Scid mice. The protection was induced only when the DNCD3 splenocytes were parked for 1 month prior to co-infusion of diabetogenic cells in the NOD/Scid mice. The results of this study supported the notion that the regulatory function of DNCD3 cells can fully develop upon differentiation in the spleen and pancreas of NOD/Scid immunodeficient mice and pre-diabetic NOD mice. DNCD3 peripheral cells from other mouse strains were previously shown to proliferate *in vitro* and *in vivo* [21], [42]. Interesting enough, the rate of DNCD3 cell proliferation in pancreas was 3 to 5 times faster in pre-diabetic NOD males than females, which is intriguingly consistent with a 2 to 3-times lower incidence of diabetes in NOD males.

DNCD3 splenic cells from young NOD mice were able to differentiate preferentially into IL-10-secreting CD4^+^ T_R_-1 like cells under Th2 stimulation. *In vivo* neutralization of IL-10 abrogated the transfer of disease in NOD/Scid mice, which clearly indicated that the anti-diabetogenic effect of differentiated DNCD3 splenic cells relied on IL-10 secretion. The ability of peripheral DNCD3 cells from other mouse strains to secrete IL-10 was previously reported [63], and we have previously demonstrated that the IL-10-secreting T_R_1-like cells prevent the T1D onset in a double transgenic mouse model [64]. There was no evidence of NOD DNCD3 splenic cell differentiation into conventional CD4^+^25^high^ T-reg cells, as the expression of CD25^high^, iCTLA-4, and Foxp3 biomarkers was absent in their quiescent state or after *in vitro* stimulation under various conditions. The DNCD3 cells were detected in the pancreatic infiltrating lymphocytes from pre-diabetic, adult NOD mice with a similar pattern of CD44/CD25, RAG2, and pTCRα expression to that of DNCD3 thymocytes. The pancreatic infiltrating lymphocytes also showed the presence of a significant number of CD3^+^(4^+^8^+^) double positive T-cells. A higher number of CD3^+^(4^+^8^+^) double positive T-cells in the pancreas than in the spleen of pre-diabetic mice may account for the presence of higher amounts of auto-antigens in the pancreas. Although the antigen does not seem to play a role in DNCD3 thymic cell differentiation, previous reports indicate that proliferation of DNCD3 splenocytes in peripheral lymphoid organs requires the presence of antigen and IL-2 [12], [19]. Together, these data argue for plasticity in the genetic program of peripheral DNCD3 cells from young NOD mice that allows them to differentiate in an extra-thymic environment into mature T-cells with regulatory (anti-diabetogenic) function. Recent data also suggest that the DNCD3 cells can develop extra-thymically in some mouse strains and humans [65].

DNCD3 splenic population from young NOD mice consisted in a majority of CD3^+^(4^−^8^−^) double negative TCRαβ cells and a small fraction of CD3^+^(4^−^8^−^) double negative TCRγδ^+^ cells (1–2%) and NKT cells (0.2–0.4%). Several arguments inferred the conclusion that the long-lasting anti-diabetogenic effect of NOD DNCD3 splenic cells was provided by the CD3^+^(4^−^8^−^) double negative TCRαβ^+^ cells rather than CD3^+^(4^−^8^−^) double negative TCRγδ^+^ cells or NKT cells. Reports showed that the intraepithelial CD3^+^(4^+^8^−^) single positive TCRγδ^+^ cells exert a suppressogenic effect on the conventional T-cells [66], but the role of peripheral CD3^+^(4^−^8^−^) double negative TCRγδ^+^ and CD3^+^(4^+^8^−^) single positive TCRγδ^+^ cells in autoimmune diabetes is still controversial [67], since the TCRγδ^+^ cells associated with either progression of diabetes [66]–[73] or protection against diabetes [39]–[41], [55]–[57], [74]. Our adoptive cell transfer experiments in NOD/Scid mice using NOD TCRγδ-depleted, DNCD3 splenic cells from young NOD mice showed a lack of anti-diabetogenic effect of CD3^+^(4^−^8^−^) double negative TCRγδ^+^ splenic cells. The role of NK cells appears to be also unrelated to autoimmune diabetes [53], [54]. Recent data suggest that iNKT cells may even exacerbate diabetes in mice [75]. Our data showed that a small number of NK/NKT cells within the DNCD3 splenic preparations from young NOD mice lacked anti-diabetogenic effect when tested in a diabetes transfer system like the NOD/Scid mouse.

An important question that remains to be addressed is why the DNCD3 regulatory cells allow development of diabetes in adult NOD mice. One possible explanation is that differentiation of DNCD3 splenic cells into anti-diabetogenic T_R_-1-like cells is greatly impaired by a gradual expansion of Th1 autoreactive cells. However, only 60–70% of NOD females and 20–40% of NOD males develop diabetes in adulthood, and this coincides with a decline of DNCD3 splenic cells in adulthood, which suggests that at least in part, individual variations in the number and/or suppressogenic threshold of DNCD3 peripheral cells may also play a role.

In summary, this study delineates a new cell population of regulatory cells (DNCD3 splenic cells) in young NOD mice with potential anti-diabetogenic effect. The phenotype of DNCD3 splenic cells is CD3^+^(CD4^−^CD8^−^)CD28^+^CD69^+^CD25^low^Foxp3^−^iCTA-4^−^TCRαβ^+^ with a predominant Vβ13 gene usage. Their suppressive (anti-diabetogenic) effect relied mainly on the ability to differentiate into IL-10-secreting T_R_-1 cells in a Th2-like extra-thymic environment. Future technologies may allow expansion of DNCD3 regulatory cells *in vitro* and provide rational grounds for the development of new autologous cell-therapies in type 1 diabetes.

## Supporting Information

Figure S1 FACS-sorting of DNCD3 splenic cells from NOD mice. Single DNCD3 cell suspensions from the spleen of NOD and NON.NOD mice isolated from a pool of 14 day-old animals (n = 20) were stained with a combination of 2 µg/10^6^ cells of CD4/CD8 Ab-PE and CD3 Ab-APC conjugates. Gated-live cells (*left panel A*) were sorted for the CD3+4−8- population (P2 window) in a FACSAria instrument at 50,000 cell events/min, and re-sorted under the same conditions (P2 window in *panel B*) to higher than 98% purity. Shown is one of two representative experiments.
(1.34 MB TIF)

Figure S2 In Vivo cell cycle division of NOD CD4-8-TCRγδ + splenic cells. A group of NOD/Scid mice (n = 6) was infused with FACS-sorted DNCD3 splenocytes (5×10^5^ cells/mouse) isolated from 14 day-old NOD females followed by CFSE injection. Seven days later, the CFSE+ cells isolated from pooled spleens of CFSE-labeled NOD/Scid recipients were stained with a combination of CD4/CD8 Ab-APC and TCRγδ Ab-PE conjugates (2 µg Ab/10^6^ cells), and the CFSE dilution factor measured within the CD4-8-TCRγδ-gated population. Shown is the majority of non dividing CD4-8- double negative TCRγδ+ cells (dark histogram) as compared with non dividing NOD DNCD3 control splenic cells labeled *in vitro* with CFSE (dotted histogram).
(1.39 MB TIF)

Figure S3 In vivo proliferation of DNCD3 splenic cells from NOD mice at various time-points after birth. Groups of young NOD littermates of 2, 7, 14, and 28 days of age (n = 3−7 mice per group) were injected intravenously (i.v.) with 0.1 mg CFSE per gram of body weight, and seven days later the spleen cells were harvested, cells from each group were pooled, and stained with CD4 Ab-PerCP Cy5.5, CD8 Ab-PE, and CD3 Ab-APC conjugates. The cell cycle divisions of CFSE+, CD3+4−8- cells (DNCD3 cells) was determined based on CFSE dilution factor in FACS using a LSR II instrument (BD Biosciences). The number of cell cycle divisions was analyzed using the WINlist software 3D 5.0. Shown are the cycles of cell division of CFSE-labeled DNCD3 splenocytes. CFSE serial dilutions of cells labeled in vitro (lower panel) indicate the number of cell cycle divisions.
(1.89 MB TIF)

Figure S4 Effect of collagenase method for isolation of lymphocytes on the CD4/CD8 phenotype. Total thymocytes from individual mice were treated in vitro with collagenase preparation as described, then washed in PBS, stained with CD4 and CD8 mAb-dye conjugates, and analyzed by FACS. Shown is a significant loss of CD4 and CD8 surface expression in one of two representative experiments.
(1.61 MB TIF)

Figure S5 Lack of Foxp3 mRNA expression in NOD DNCD3 splenic cells. FACS-sorted DNCD3 splenic cells (10^6^ cells), and negatively-sorted mature splenic CD4+ T-cells (10^6^ control cells) were isolated from 14 day-old female mice (n = 10) and stimulated for 5 days under Th1 or Th2 conditions, or for 1 day under T-reg conditions (2.5 µg/ml of CD3/CD28 mAb and TGF-β, as described. mRNA was amplified in RT-PCR using specific primers for Foxp3. Lane 1, molecular markers; lane 2, DN cells stimulated under Th1 conditions; lane 3, DN cells stimulated under Th2 conditions; lane 4, DN cells stimulated with CD3/CD28 mAbs and TGF-β, and lane 5, splenic CD4+ mature T-cells stimulated with CD3/CD28 mAbs alone. Foxp3 transcript was detected only in splenic CD4+ mature T-cells. Lower panel shows the GAPDH mRNA amplicons corresponding to each sample analyzed in the upper panel.
(1.24 MB TIF)
