# Supplementary figures and images for: Double Negative (CD3+4−8−) TCRαβ Splenic Cells from Young NOD Mice Provide Long-Lasting Protection against Type 1 Diabetes
Source: PLoS One. 2010 Jul 2;5(7):e11427. doi: 10.1371/journal.pone.0011427 (PMC2896421; doi:10.1371/journal.pone.0011427)

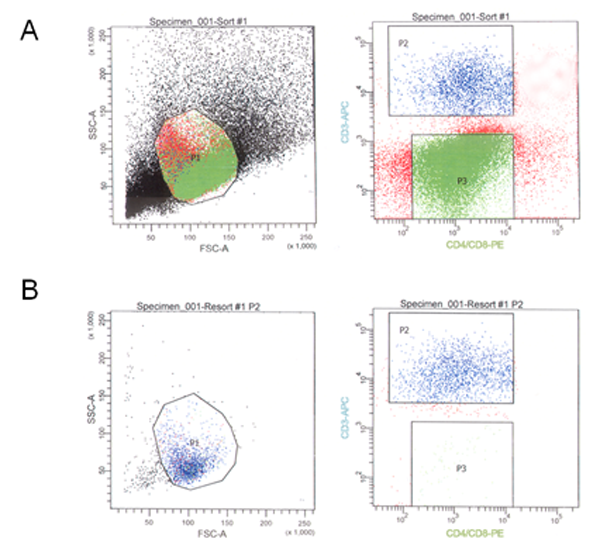

Supplement: Figure S1 — FACS-sorting of DNCD3 splenic cells from NOD mice. Single DNCD3 cell suspensions from the spleen of NOD and NON.NOD mice isolated from a pool of 14 day-old animals (n = 20) were stained with a combination of 2 µg/106 cells of CD4/CD8 Ab-PE and CD3 Ab-APC conjugates. Gated-live cells (left panel A) were sorted for the CD3+4−8- population (P2 window) in a FACSAria instrument at 50,000 cell events/min, and re-sorted under the same conditions (P2 window in panel B) to higher than 98% purity. Shown is one of two representative experiments. (1.34 MB TIF) [file pone.0011427.s001.tif]

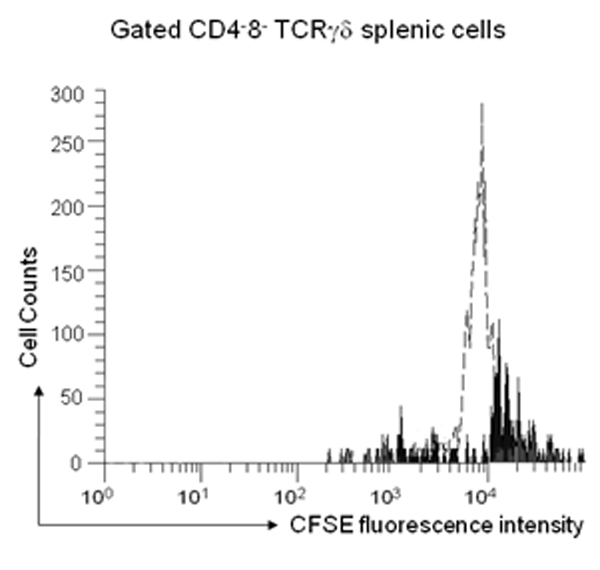

Supplement: Figure S2 — In Vivo cell cycle division of NOD CD4-8-TCRγδ + splenic cells. A group of NOD/Scid mice (n = 6) was infused with FACS-sorted DNCD3 splenocytes (5×10^5^ cells/mouse) isolated from 14 day-old NOD females followed by CFSE injection. Seven days later, the CFSE+ cells isolated from pooled spleens of CFSE-labeled NOD/Scid recipients were stained with a combination of CD4/CD8 Ab-APC and TCRγδ Ab-PE conjugates (2 µg Ab/106 cells), and the CFSE dilution factor measured within the CD4-8-TCRγδ-gated population. Shown is the majority of non dividing CD4-8- double negative TCRγδ+ cells (dark histogram) as compared with non dividing NOD DNCD3 control splenic cells labeled in vitro with CFSE (dotted histogram). (1.39 MB TIF) [file pone.0011427.s002.tif]

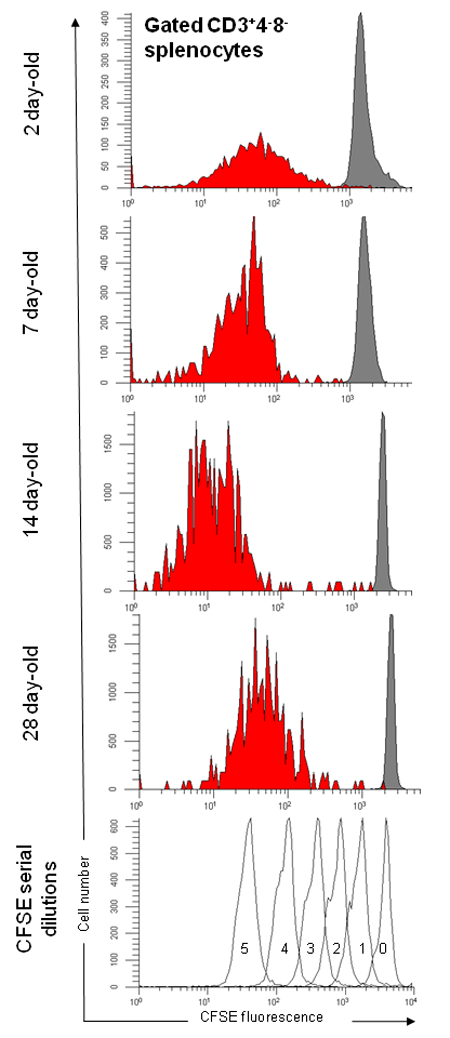

Supplement: Figure S3 — In vivo proliferation of DNCD3 splenic cells from NOD mice at various time-points after birth. Groups of young NOD littermates of 2, 7, 14, and 28 days of age (n = 3−7 mice per group) were injected intravenously (i.v.) with 0.1 mg CFSE per gram of body weight, and seven days later the spleen cells were harvested, cells from each group were pooled, and stained with CD4 Ab-PerCP Cy5.5, CD8 Ab-PE, and CD3 Ab-APC conjugates. The cell cycle divisions of CFSE+, CD3+4−8- cells (DNCD3 cells) was determined based on CFSE dilution factor in FACS using a LSR II instrument (BD Biosciences). The number of cell cycle divisions was analyzed using the WINlist software 3D 5.0. Shown are the cycles of cell division of CFSE-labeled DNCD3 splenocytes. CFSE serial dilutions of cells labeled in vitro (lower panel) indicate the number of cell cycle divisions. (1.89 MB TIF) [file pone.0011427.s003.tif]

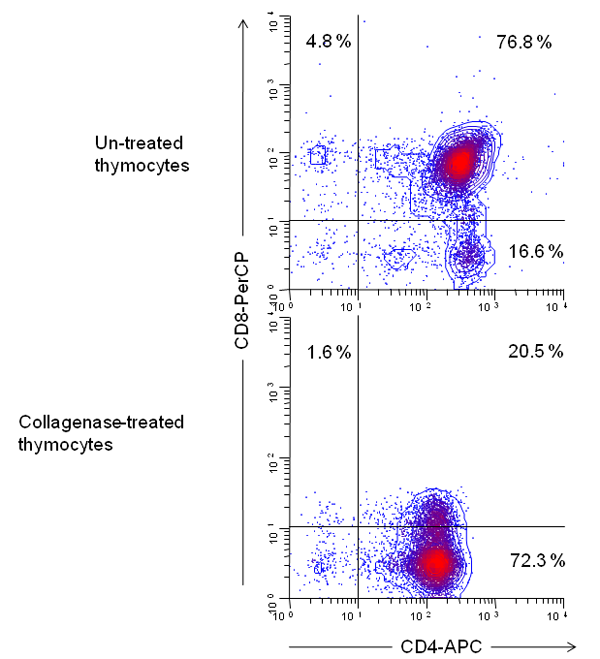

Supplement: Figure S4 — Effect of collagenase method for isolation of lymphocytes on the CD4/CD8 phenotype. Total thymocytes from individual mice were treated in vitro with collagenase preparation as described, then washed in PBS, stained with CD4 and CD8 mAb-dye conjugates, and analyzed by FACS. Shown is a significant loss of CD4 and CD8 surface expression in one of two representative experiments. (1.61 MB TIF) [file pone.0011427.s004.tif]

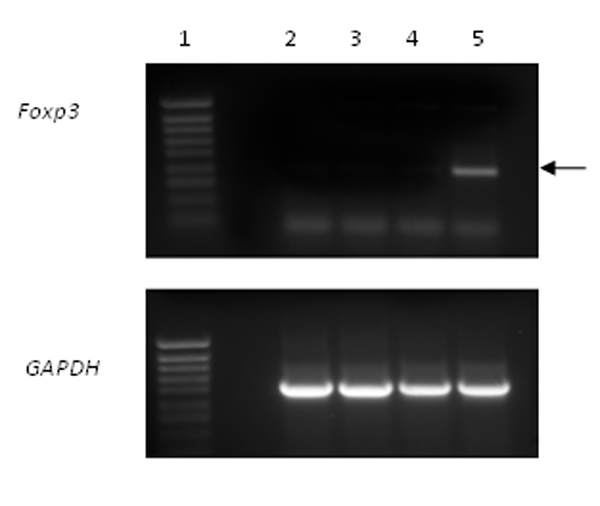

Supplement: Figure S5 — Lack of Foxp3 mRNA expression in NOD DNCD3 splenic cells. FACS-sorted DNCD3 splenic cells (106 cells), and negatively-sorted mature splenic CD4+ T-cells (106 control cells) were isolated from 14 day-old female mice (n = 10) and stimulated for 5 days under Th1 or Th2 conditions, or for 1 day under T-reg conditions (2.5 µg/ml of CD3/CD28 mAb and TGF-β, as described. mRNA was amplified in RT-PCR using specific primers for Foxp3. Lane 1, molecular markers; lane 2, DN cells stimulated under Th1 conditions; lane 3, DN cells stimulated under Th2 conditions; lane 4, DN cells stimulated with CD3/CD28 mAbs and TGF-β, and lane 5, splenic CD4+ mature T-cells stimulated with CD3/CD28 mAbs alone. Foxp3 transcript was detected only in splenic CD4+ mature T-cells. Lower panel shows the GAPDH mRNA amplicons corresponding to each sample analyzed in the upper panel. (1.24 MB TIF) [file pone.0011427.s005.tif]
